# Method Development and Validation of a Stability-Indicating RP-HPLC Method for the Quantitative Analysis of Dronedarone Hydrochloride in Pharmaceutical Tablets

**DOI:** 10.3797/scipharm.1209-15

**Published:** 2012-11-05

**Authors:** Batuk Dabhi, Yashwantsinh Jadeja, Madhavi Patel, Hetal Jebaliya, Denish Karia, Anamik Shah

**Affiliations:** 1 Department of Chemistry, Saurashtra University, Rajkot-360 005, Gujarat, India.; 2 Arts Commerce and Science College, Borsad, Anand, Gujarat, India.

**Keywords:** Dronedarone Hydrochloride, Stability-indicating, HPLC

## Abstract

A simple, precise, and accurate HPLC method has been developed and validated for the quantitative analysis of Dronedarone Hydrochloride in tablet form. An isocratic separation was achieved using a Waters Symmetry C_8_ (100 × 4.6 mm), 5 μm particle size column with a flow rate of 1 ml/min and UV detector at 290 nm. The mobile phase consisted of buffer: methanol (40:60 v/v) (buffer: 50 mM KH_2_PO_4_ + 1 ml triethylamine in 1 liter water, pH=2.5 adjusted with ortho-phosphoric acid). The method was validated for specificity, linearity, precision, accuracy, robustness, and solution stability. The specificity of the method was determined by assessing interference from the placebo and by stress testing the drug (forced degradation). The method was linear over the concentration range 20–80 μg/ml (r^2^ = 0.999) with a Limit of Detection (LOD) and Limit of Quantitation (LOQ) of 0.1 and 0.3 μg/ml respectively. The accuracy of the method was between 99.2–100.5%. The method was found to be robust and suitable for the quantitative analysis of Dronedarone Hydrochloride in a tablet formulation. Degradation products resulting from the stress studies did not interfere with the detection of Dronedarone Hydrochloride so the assay is thus stability-indicating.

## Introduction

**Fig. 1:**
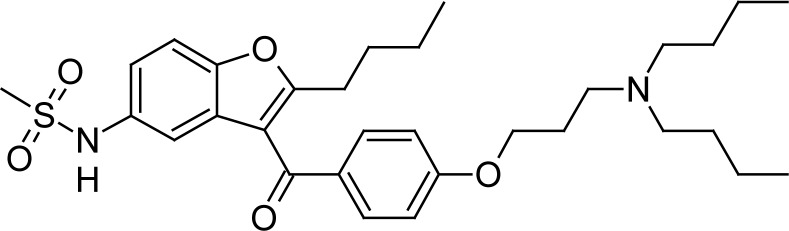
Structure of Dronedarone

Dronedarone hydrochloride, mainly used for the treatment of cardiac arrhythmias, is chemically *N*-(2-butyl-3-{4-[3-(dibutylamino)propoxy]benzoyl}-1-benzofuran-5-yl)methane-sulfonamide (Fig. 1). Its molecular formula is C_31_H_44_N_2_O_5_ HCl and molecular weight is 556.76. The drug is approved to be used in patients whose hearts have returned to normal rhythm or who will undergo drug or electric-shock treatment to restore a normal heart beat [1].

High-performance Liquid Chromatography (HPLC) is a well-known and widely used analytical technique for the analysis of drug products and drug substances. Some articles exist about the analysis of dronedarone in human plasma by liquid chromatography-tandem mass spectrometry [2], the combination with amiodarone and their principle metabolites in plasma and myocardium by HPLC and UV-Detection [3], in bulk drugs by HPLC [4], and for the stability-indicating analysis by HPLC [5]. The objective of this work was to develop a stability-indicating liquid chromatographic analytical method for the assay of dronedarone hydrochloride in a tablet formulation. The validation procedure followed the guidelines of the ICH (International Conference on Harmonisation of Technical Requirements for Registration of Pharmaceuticals for Human Use) [6] and the USP (United States Pharmacopeia) [7].

## Material and Methods

The dronedarone hydrochloride reference standard (claim 99.48%) was provided by Sanofi-Aventis. Tablets of dronedarone hydrochloride (400 mg) were purchased from a pharmacy. HPLC grade methanol and orthophosphoric acid were obtained from Merck India Limited, Mumbai, India. Analytical grade hydrochloric acid, sodium hydroxide pellets, and hydrogen peroxide solution 30% (v/v) were obtained from Ranbaxy Fine Chemicals, New Delhi, India, and a 0.45 μm membrane filter was obtained from Pall Life Sciences, Mumbai, India. High purity deionised water was obtained from a Milli-Q (Millipore, Milford, MA, USA) purification system. Nylon syringe filters 0.45 μm were from Millex-Hn (Mumbai, India).

### Chromatography

Liquid chromatography was performed with Waters HPLC equipment with a TM 600 quaternary pump, Waters 2489 uv/vis detector, Waters 600 controller, Waters in-line degasser AF, and manual injector with a 20 μL loop. The equipment was connected to a multi-instrument data-acquisition and data-processing system (Empower software). The chromatographic system was performed using a Waters Symmetry C_8_ (100 × 4.6mm i.d.), 5μm column. Separation was achieved using a mobile phase consisting of buffer: methanol (40:60 v/v) (buffer: 50 mM KH_2_PO_4_ + 1 ml triethylamine in 1 liter water, pH=2.5 adjusted with orthophosphoric acid) at a flow rate of 1 ml/min with a short runtime (12 min). The eluent was monitored using UV detection at a wavelength of 290 nm. The column temperature was maintained at 30 °C and the injection volume 20 μL was used. The mobile phase was filtered through a 0.45 μm micron filter prior to use.

#### Preparation of sample solution

To prepare a stock solution (500 μg/ml) for the assay, 10 tablets were weighed and mixed. An aliquot of powder equivalent to the weight of five tablets was accurately weighed and transferred to a 50 ml volumetric flask and dissolved in 25 ml of methanol and the mixture was sonicated for 30 min. The contents of the flask were then left to return to room temperature and the volume was adjusted with the water: methanol (40:60 v/v). Solution was then filtered through a 0.45 μm nylon syringe filter.

To prepare the test solution of 50 μg/ml for the assay, 5 ml of test stock solution was transferred to a 50 ml volumetric flask and the volume was adjusted with water: methanol (40:60, v/v) and shaken well.

## Result and Discussion

### Method Development

During the process of method development, several trials were taken using a different buffer, different column, different organic phase, and a different pH of the buffer. Good peak shape was observed when using the Waters Symmetry C_8_ (100:4.6 mm, 5u) column and 50 mM KH_2_PO_4_ + 1 mL of triethylamine (pH=2.5 by orthophosphoric acid): methanol (40:60) as the mobile phase at a flow rate of 1ml/min.

The chromatographic conditions were optimized to separate all of the possible degradation impurities from the peak of dronedarone hydrochloride in the short runtime. The drug substance was easily extracted from the pharmaceutical dosage form using methanol. The drug substance was freely soluble in methanol. Solutions of the standard and test preparation were found to be stable in water:methanol (40:60 v/v).

### Method Validation

Accuracy, precision, linearity, selectivity, robustness, Limit of Detection (LOD), LOQ (Limit of Quantification), and system suitability were performed as a method validation parameter as per ICH guidelines.

#### Specificity

The specificity of the method was evaluated to ensure there was no interference from the placebo components (prepared in solution) or from products resulting from forced degradation.

#### Forced degradation studies

**Fig. 2:**
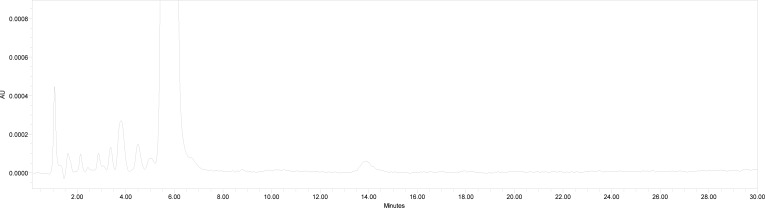
Acid Degradation Study of Dronedarone Hydrochloride

**Fig. 3:**
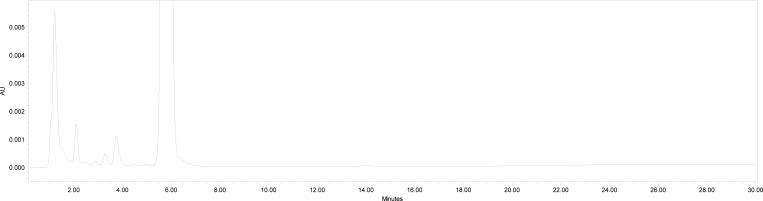
Base Degradation Study of Dronedarone Hydrochloride

**Fig. 4:**
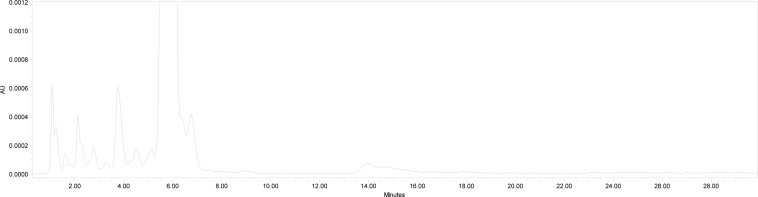
H_2_O_2_ Degradation Study of Dronedarone Hydrochloride

**Fig. 5:**
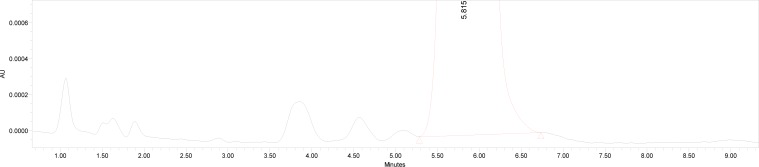
Thermal Degradation Study of Dronedarone Hydrochloride

**Fig. 6:**
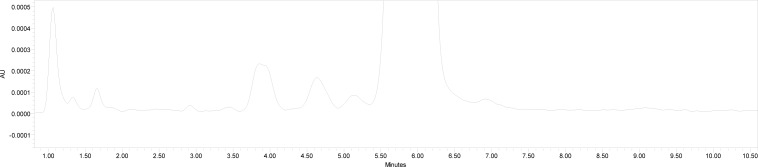
Photo Degradation Study of Dronedarone

The degradation study was performed to ensure that the method was able to separate dronedarone from the probable degradation products generated during the forced degradeation study. Acid, base, oxidative, sunlight, and thermal degradation studies were performed. Acidic degradation of the drug was performed by heating the drug with 1M HCl for 2 hrs at 80 °C. Alkaline degradation was performed by heating the drug with 0.1 M NaOH for 2 hrs at 80 °C. Oxidizing degradation was performed by heating the drug with 6% v/v H_2_O_2_ at 80 °for 2 hrs. Thermal degradation was performed by heating the drug at 70 °C for 48 hrs. Powdered drug was exposed to sunlight for 48 hrs for photolytic degradation. The placebo was also subjected to the same stress conditions to identify any response due to the forced degradation conditions. Solutions were then left to return to room temperature and were diluted with water:methanol (40:60) to furnish 50 μg/ml of solution. Major degradation up to 12% occurred under acidic conditions (Fig. 2), 32% under alkaline conditions (Fig. 3), 16% under oxidizing conditions (Fig. 4), 28% under thermal degradation (Fig. 5), and 8% degradation occurred under photolytic conditions (Fig. 6).

The intensive approach described in this manuscript was to develop and validate a liquid chromatographic analytical method that can be used for the assay of dronedarone hydrochloride in a pharmaceutical dosage form. The degradation products produced as a result of stress did not interfere with the detection of dronedarone hydrochloride, and the assay method can thus be regarded as stability-indicating.

#### Linearity

**Fig. 7:**
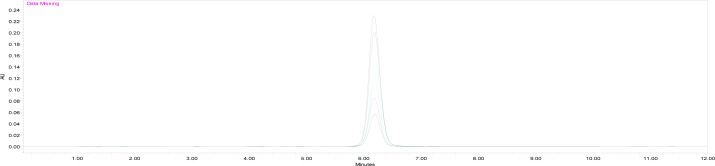
Linearity Study of Dronedarone Hydrochloride

Eight solutions were prepared containing 20, 30, 40,50, 60, 70, and 80 μg/ml dronedarone hydrochloride concentration which corresponded to 40%, 60%, 80%, 100%, 120%, 140%, and 160% respectively (Fig. 7). Each solution was injected in duplicate. Linearity was evaluated by linear-regression analysis which was obtained by plotting the dronedarone hydrochloride concentration against the peak area. The correlation coefficient was found to be 0.999. The results of linearity, Limit of Detection, and Limit of Quantification parameters are shown in Table 1.

#### Precision

System precision was evaluated by analyzing the standard solution five times, and the method precision (repeatability) was evaluated by assaying six sets of test samples prepared for the determination, all on the same day (intra-day precision). System precision and method precision were also determined by performing the same procedures on a different day (inter-day precision), and by another person under the same experimental conditions (intermediate precision). Precision was determined by calculating % RSD. For the assay (n=6), % RSD for the system precision was 0.64% on the same days (intra-day) and 0.41% on different days (inter-day).

#### Accuracy

Accuracy was assessed by the determination of the recovery of the method at three different concentrations (corresponding to 50, 100, and 150% of test solution concentrations) by the addition of known amounts of the standard to the placebo preparation. Three sets were prepared for each concentration and injected in duplicate. The accuracy of the method was assessed by the determination of recovery for the three concentrations covering the range of the method. The mean recovery of Dronedarone Hydrochloride was between 99.2–100.5%, which is satisfactory (Table 2).

#### Robustness

The robustness of the method was evaluated by assaying the test solutions after slight but deliberate changes in the analytical conditions. The factors chosen for this study were the flow rate (±0.1 ml/min.), mobile phase composition (methanol:buffer, 62:38, v/v) and (methanol:buffer, 58:42), and using another HPLC column. The standard solution and test solution were prepared separately for each different analytical condition. The results obtained from the assay of the test solution were not affected by the varying conditions and were in accordance with the true value. System suitability data were also found to be satisfactory during the variation of the analytical conditions. The analytical method therefore remained unaffected by slight but deliberate changes in the analytical conditions (Table 3).

#### Solution stability

Stability in solution was evaluated by the standard solution and the test preparation. The solution was stored at 5 °C at ambient temperature without protection from light and tested after 12, 24, 36, and 48 hrs. The responses for the aged solution were evaluated by comparison with freshly prepared solutions. The stability study of the stored standard solution and test preparation were performed and solutions were found to be stable for up to 48 hrs. The assay values obtained after 36 hr. were statistically identical with the initial value without measurable loss.

#### System suitability

The suitability of the chromatographic system was tested before each stage of validation. Five replicate injections of the standard preparation were injected and the asymmetry, number of theoretical plates, and relative standard deviation of the peak area were determined.

## Figures and Tables

**Tab. 1.** Result of Linearity, LOD, and LOQ Study of Dronedarone

**Parameter**	**Dronedarone**
Linearity (μg/mL)	20–80
Correlation coefficient (r^2^)	0.999
LOD (μg/mL)	0.10
LOQ (μg/mL)	0.30

**Tab. 2.** Accuracy Study of Dronedarone

**Level %**	**No**	**Amount of drug**		**Recovery (%)**	**Mean Recovery (%)**	**RSD (%)**
		**added (μg/ml)**	**found (μg/ml)**			
50	1	24.56	25.68	100.48	100.43	0.2612
	2	25.12	25.27	100.59		
	3	25.18	25.76	101.21		

100	1	50.46	50.09	99.27	99.22	0.1578
	2	49.79	49.46	99.35		
	3	50.43	49.93	99.04		

150	1	75.68	76.11	100.58	100.54	0.0845
	2	74.67	74.67	100.56		
	3	75.28	75.65	100.49		

**Tab. 3.** Robustness Study of Dronedarone

**Conditions**	**Assay (%)**	**RT (min)**	**System suitability data**	
			**Theoretical Plates**	**Asymmetry**
0.9 ml/min flow	99.02	5.7	5650	1.29
1.1 ml/min flow	99.12	7.1	5274	1.25
Buffer:Mehanol (42:58 v/v)	99.23	8.3	5359	1.20
Buffer:Methanol (38:62v/v)	98.89	4.9	5284	1.25
Column (Lot Change)	99.32	6.2	5642	1.26

**Tab. 4.** Comparison of developed method with published methods

**Parameter**	**Method of Ref. 4**	**Method of Ref. 5**	**Proposed Method**
Runtime	Description shows 7 min but in chromatogram runtime is 10 min	25 min	12 min
Stability Indicating	No	Yes	Yes
Isocratic/Gradient	Isocratic	Gradient	Isocratic
LOD	0.87 μg/mL	Not available for dronedarone	0.1 μg/mL
LOQ	2.65 μg/mL	Not available for dronedarone	0.3 μg/mL
